# Impact of Neoadjuvant Chemotherapy on Immediate Breast Reconstruction: A Meta-Analysis

**DOI:** 10.1371/journal.pone.0098225

**Published:** 2014-05-30

**Authors:** Junlong Song, Xiang Zhang, Qiang Liu, Jianheng Peng, Xinjie Liang, Yuanyuan Shen, Hongtao Liu, Hongyuan Li

**Affiliations:** 1 Department of Endocrine and Breast Surgery, The First Affiliated Hospital of Chongqing Medical University, Chongqing, China; 2 Department of General Surgery, The People’s Hospital of Dazu County, Chongqing, China; IRCCS National Cancer Institute, Italy

## Abstract

**Objective:**

The objective of this study was to perform a meta-analysis of published studies for evaluating the impact of neoadjuvant chemotherapy (NAC) on immediate breast reconstruction.

**Methods:**

We searched medical databases to identify appropriate studies that assessed the impact of NAC on immediate breast reconstruction from the inception of this technique through April 2013. We then performed a meta-analysis of these studies.

**Results:**

Our searches identified 11 studies among 1,840 citations. In the meta-analysis, NAC did not increase the overall rate of complications after immediate breast reconstruction (odds ratio [OR] = 0.59; 95% confidence interval[CI] = 0.38–0.91). The complication rate was also unaffected by NAC when we considered infections (OR = 0.82; 95% CI = 0.46–1.45), hematomas (OR = 1.35; 95% CI = 0.57–3.21), and seromas (OR = 0.77; 95% CI = 0.23–2.55). Additionally, expander or implant loss did not significantly increase in patients after NAC (OR = 1.59; 95% CI = 0.91–2.79). Only 2 studies (202 procedures) had reported total autologous flap loss, and they were included in our analysis; both studies found no association between NAC and total flap loss.

**Conclusion:**

Our analysis suggests that NAC does not increase the complication rate after immediate breast reconstruction. For appropriately selected patients, immediate breast reconstruction following NAC is a safe procedure. The best way to study this issue in the future is to conduct a multicenter prospective study with a longer follow-up period and more clearly defined parameters.

## Introduction

Breast conservation surgery, which has become popular in recent years, provides effective locoregional management and improved quality of life when compared with a mastectomy [1]. However, many patients still require mastectomies as standard treatment for breast cancer. Many of these patients choose to undergo immediate breast reconstruction [2]. Breast reconstruction is playing an increasingly significant role in the treatment of breast cancer. There are 3 main types of reconstruction performed on these patients. Patients may undergo autogenous reconstruction, which uses the autogenous tissue alone to perform reconstruction; expander/implant (E/I) reconstruction, which uses expanders or implants to replace the removed breast tissue; or a third approach, which uses both autogenous tissue and E/I for reconstruction. Breast reconstruction can be performed immediately or after the patient recovers from breast cancer surgery, which is called delayed breast reconstruction. Patients who receive immediate reconstruction have better aesthetic results, better psychosocial outcomes, and lower costs of surgery compared to patients who undergo delayed reconstruction or no reconstruction [3]. Furthermore, many studies have shown that immediate breast reconstruction does not increase local or distant recurrence, demonstrating the oncological safety of this technique [4]–[7]. Immediate breast reconstruction after mastectomy is now routinely recommended for appropriate patients according to the National Comprehensive Cancer Network guidelines [8].

Neoadjuvant chemotherapy (NAC) is defined as adjuvant systemic therapy that is administered prior to, rather than following, locoregional treatment. NAC was first introduced for the management of breast cancer approximately 30 years ago [9], [10]. From the time of its introduction, NAC has not only been administered to patients with later-stage cancer to downstage their disease but has also been administered to patients to increase the feasibility of breast conserving surgery; it has also been used to examine the response of the tumor to the chemotherapy regimen [11], [12].

However, as nearly all NAC agents are cytotoxic, they may theoretically affect surgical outcomes by causing complications such as infections and problems with wound healing. We wanted to discern whether NAC increases the rate of complications after immediate breast reconstruction. To address this issue, we performed a meta-analysis to integrate the results from recent studies that have examined the influence of NAC on immediate breast reconstruction after mastectomies.

## Methods

### Data Sources

We searched the literature by using PubMed, EMBASE, Google Scholar, the Cochrane Library, the China National Knowledge Infrastructure whole article database, and the VIP Chinese Journals Database to identify studies about the influence of NAC on immediate breast reconstruction after mastectomies. The following terms were used when searching for articles: “preoperative chemotherapy,” “neoadjuvant chemotherapy,” “breast reconstruction,” “outcomes,” and “complications.” We also manually searched the available of journals in our library. Unpublished studies were not included in our analysis. No language restrictions were applied to the search. The most recent search was performed on April 1, 2013. The 2 authors (Song and Li) independently examined the titles and abstracts of citations, and they obtained the full text of potentially eligible trials. Disagreements between the authors were resolved by discussion. If a patient cohort was reported more than once, the most informative and recent study with complete data was chosen.

### Inclusion Criteria

To be included in our analysis, studies had to meet the following criteria: study patients who underwent immediate breast reconstruction after mastectomies to remove breast cancer; the study had to include intervention with preoperative chemotherapy and a control group, report at least 1 outcome mentioned in the Definitions section of this paper, and have a sufficiently long follow-up period (for example, 30 days). Studies could be designed as randomized control trials (RCTs) or a non-RCT.

### Exclusion Criteria

Studies were excluded from our analysis if they did not include a control group, did not have extractable data, or were case reports or reviews.

### Definitions

Flap loss was defined as a total loss of circulation in the autologous flap. An infection was defined as localized or systemic evidence of infection that led to administration of oral antibiotics or hospital admission for intravenous antibiotics. A hematoma was defined as a collection of blood at the surgical area that required surgical treatment. A seroma was defined as a clinically obvious collection of serous fluid at the surgical area that required aspiration.

### Quality Score

We used the Newcastle-Ottawa Scale (NOS) [13], [14] to assess the quality of the studies included in our analysis, because the studies were all non-randomized. The 2 authors (Song and Li) independently assessed the included studies and disagreements were resolved by discussion.

### Data Extraction

The following information was extracted from each eligible trial: authors’ names, country, date, number of patients, method of reconstruction, NAC regimen, and follow-up period (see Tables 1, 2).

**Table 1:** Demographic characteristics and quality scores of studies included in the analysis.

Author	Years ofdiagnosis	Country	Patients(NAC/Control)	Age(range; years)	NAC regimens	Follow-up	Reconstructiontype	Outcomes reported	NOSscore
Donker, 2011	2006–2009	Netherlands	48/215	NAC: median 38 (20–62);Control: median 47 (25–70)	AC;Capecitabine+T	6 weeks	E/I	Seroma, hematoma, infection,skin necrosis, surgicalre-intervention, implant loss	8
Jiménez-Puente,2011	2002–2009	Spain	13/102	NA	FEC; AC-T	9 months	E/I	Infection, seroma, dehiscence,hemorrhage, mechanicalcomplication, extrusion, necrosis	6
Radovanovic, 2010	2004–2008	Serbia	42/172	mean 47 (26–69)	NA	6 weeks	E/I	Epidermolysis, infection,skin necrosis, seromaformation, hematoma	6
Schaverien, 2013	2006–2012	UK	57/65	NAC: mean 46.5 (35–54);Control: mean 49 (33–70)	AC-T	8–35 months	Auto	Complete flap loss, hematoma,seroma, infection, donor sitebreakdown, fat necrosis,mastectomy skin flap necrosis,hospital-acquired pneumonia	7
Azzawi, 2010	2000–2007	UK	58/140	NAC: mean 47.8 (29–68);Control: mean 50.4 (29–69)	EC-T; CMF-E;CMF; AT; AC	7–64 months	E/I, Auto	Wound infection, slow healing,wound breakdown, fat necrosis,flap loss, partial flap necrosis,hematoma, infected implant,pulmonary embolism	8
Zweifel-Schlatter,2010	2007–2009	UK	49/58	NAC: median 47 (31–68);Control: mean 49 (35–63)	FEC; AC-T	30 days	Auto	Total or partial flap loss,hematoma, infection, woundhealing problem, woundbreakdown, seroma	7
Liu, 2009	2001–2007	Japan	12/63	NAC: mean 45.3 (26–63);Control: mean 43.3 (30–57)	EC; FEC; T	NA	E/I	Flap necrosis, infection,seroma, hematoma	7
Godfrey, 1995	NA	USA	11/10	NA	NA	>8 months	Auto	Seroma, lymphocoele, mastectomyflap slough, cellulitis	5
Decker, 2012	2005–2010	USA	380/8474	NAC: mean 52.1±12.0;Control: mean 59.63±13.1	NA	30 days	NA	Wound complications (superficialsurgical site infection, deepinfection, wound dehiscence)	6
Hu, 2011	1997–2007	USA	42/214	NA	AC; TAC; T	60 days	E/I, Auto	Seroma, hematoma, surgical siteinfection, dehiscence, open wound,skin necrosis, flap loss	6
Peled, 2010	2005–2007	USA	57/65	NAC: mean 46.4 (28–71);Control: mean 49.8 (25–70)	NA	8–35 months	E/I, Auto	Infection, skin flap necrosis,flap loss, E/I loss, unplannedreturn to the operating room,donor-site complications	7

Abbreviations: Auto, autologous; E/I, expander/implant; NA, not available; NAC, neoadjuvant chemotherapy; NOS, Newcastle-Ottawa Scale. NAC regimens: A, doxorubicin; C, cyclophosphamide; E, epirubicin; F, fluorouracil; M, methotrexate; T, taxotere.

**Table 2:** Raw data showing the complications.

Author	Reconstruction type	Patients		Totalcomplications		Hematomas		Seromas		Infections		E/I loss		Totalflap loss		Reoperations	
		NAC	Control	NAC	Control	NAC	Control	NAC	Control	NAC	Control	NAC	Control	NAC	Control	NAC	Control
Donker, 2011	E/I	48	215	7/48	62/215	0/48	11/215	1/48	4/215	4/48	29/215	4/48	23/215	NA	NA	6/48	55/215
Jiménez-Puente, 2011	E/I	13	102	NA	NA	NA	NA	NA	NA	NA	NA	7/13	19/102	NA	NA	NA	NA
Radovanovic, 2010	E/I	42	172	4/42	31/172	NA	NA	NA	NA	NA	NA	3/42	9/172	NA	NA	NA	NA
Schaverien, 2013	Auto	33	62	NA	NA	NA	NA	NA	NA	1/33	1/62	NA	NA	1/33	1/62	NA	NA
Azzawi, 2010	E/I, Auto	58	140	NA	NA	0/58	1/140	NA	NA	NA	NA	NA	NA	NA	NA	5/58	13/140
Zweifel-Schlatter, 2010	Auto	49	58	NA	NA	3/49	2/58	2/49	3/58	3/49	5/58	NA	NA	2/49	1/58	NA	NA
Liu, 2009	E/I	12	63	3/12	19/63	1/12	3/63	0/12	2/63	1/12	2/63	NA	NA	NA	NA	NA	NA
Godfrey, 1995	Auto	11	10	2/11	6/10	NA	NA	0/11	1/10	NA	NA	NA	NA	NA	NA	NA	NA
Hu, 2011	E/I, Auto	42	214	15/42	80/214	NA	NA	NA	NA	NA	NA	NA	NA	NA	NA	NA	NA
Peled, 2010	E/I, Auto	57	65	NA	NA	5/57	1/65	NA	NA	13/57	16/65	8/31	8/45	NA	NA	19/57	18/65

Abbreviations: Auto, autologous; E/I, expander/implant; NA, not available; NAC, neoadjuvant chemotherapy.

### Statistical Analysis

Our meta-analysis was performed according to the recommendations of the Cochrane Collaboration, the Quality of Reporting of Meta-Analyses guidelines, and the Meta-analysis of Observational Studies in Epidemiology recommendations [15], [16]. If the heterogeneity was not obvious, the odds ratios (ORs) of postoperative complications were pooled by using a fixed effects model. Otherwise, we used a random effects model to pool the ORs. We also conducted a sensitivity analysis to examine the stability of the pooled results. The heterogeneity between ORs was assessed by using the I2 index and by performing a test to determine the overall effect (Z-statistic with P-value). The 95% CI of all ORs were calculated, and P<0.05 was considered statistically significant. Analyses were conducted by using Review Manager version 5.1 (The Cochrane Collaboration, Copenhagen, Denmark). All statistical tests were 2-tailed.

## Results

### Eligible Studies

**Figure 1:**
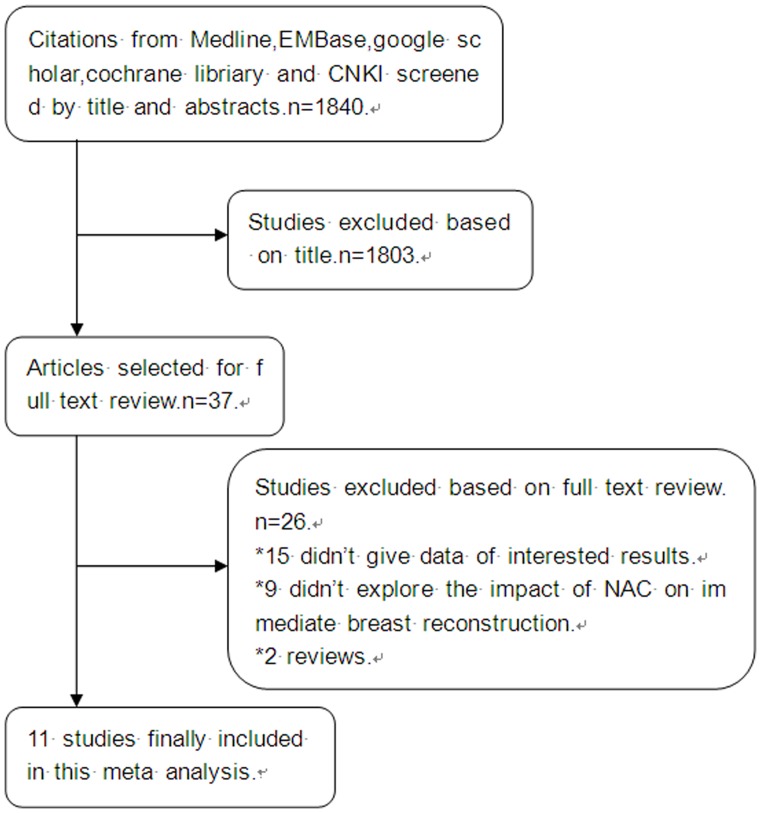
Flow chart of the study selection process.

Figure 1 illustrates the process of evaluating articles for inclusion in the review and meta-analysis. We obtained 1,840 citations from the previously mentioned databases. We examined the titles and abstracts of these references and excluded all the studies that were clearly ineligible for the analysis. For the remaining articles, we obtained full text copies for further evaluation. In the databases that we searched, we obtained 14 potentially eligible studies that examined the influence of NAC on immediate breast reconstruction [9], [11], [17]–[28]. Eleven eligible studies addressed the influence of NAC on immediate breast reconstruction, and these studies also included extractable data; they were included in the review and meta-analysis [9], [11], [17]–[23], [25], [28].

Table 1 summarizes the descriptive data for each of the studies included in this systematic review and meta-analysis. The majority of the studies were performed in the United States and United Kingdom. Most studies were published within the last 10 years. There was only 1 older study, a report by Godfrey et al. [25] published in 1995. Sample sizes ranged from 21 patients to 8,854 patients. On the basis of the methodology and reported data, the studies included in the meta-analysis were deemed to be of a moderate to high quality overall, with all of the included studies ranking ≥5 stars on the modified NOS. Ten of the studies were performed by using medical records to ascertain complications in clinic-based cohorts. One study [17] was performed by using data from the American College of Surgeons National Surgical Quality Improvement Program. Seven studies focused on NAC as the primary exposure, while the other 4 studies evaluated NAC along with several different prognostic factors. These differences in study design and outcome assessment likely produced the heterogeneity identified by performing the Cochran Q and I2 statistical analysis.

### NAC and Overall Complications

**Figure 2:**
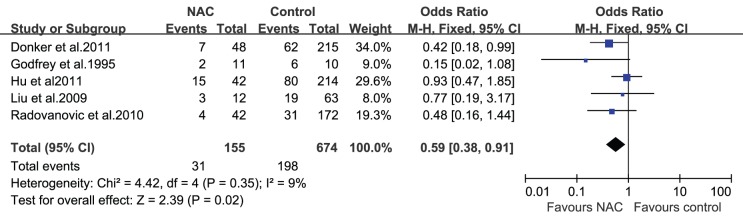
Pooled results of overall complications in patients treated with NAC. The size of the solid squares is inversely proportional to the variance of the study estimate. The diamond represents the fixed effects odds ratio and 95% confidence interval.

Of the 11 studies in the meta-analysis, 5 reported a risk estimate of NAC with respect to the overall complications. The characteristics and demographics of the selected studies, along with an estimate of precision and list of satisfied eligibility criteria for the meta-analysis, are listed in Table 1. Five studies (n = 829 patients) [9], [11], [19], [20], [25] reported the overall complication rate. Although the 5 studies showed no statistical significance individually, the pooled OR was statistically significant. When we pooled the results of these studies, NAC was associated with a decreased risk of overall complications (OR = 0.59; 95% confidence interval [CI] = 0.38–0.91; Fig. 2). In the fixed effect model, the pooled risk estimate for E/I-based reconstruction studies resulted in an OR of 0.49 (95% CI = 0.26–0.89). For studies with larger sample sizes and higher quality, the pooled OR was 0.62 (95% CI = 0.38–0.99). For studies with a short follow-up period (<6 weeks), the pooled OR was 0.44 (95% CI = 0.23–0.87). Exclusion of the study by Donker et al. resulted in the highest pooled estimate (OR = 0.67; 95% CI = 0.40–1.12), and exclusion of the study by Hu et al. resulted in the lowest pooled estimate (OR = 0.44; 95% CI = 0.25–0.79).

### NAC and Wound Complications

**Figure 3:**
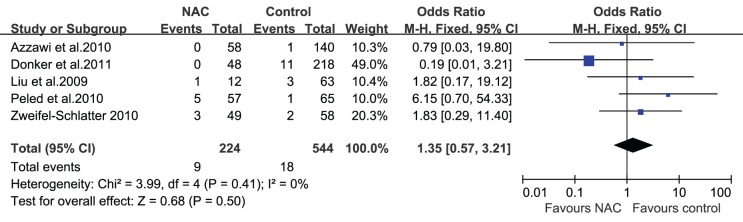
Pooled results of hematomas in patients treated with NAC. The size of the solid squares is inversely proportional to the variance of the study estimate. The diamond represents the fixed effects odds ratio and 95% confidence interval.

**Figure 4:**
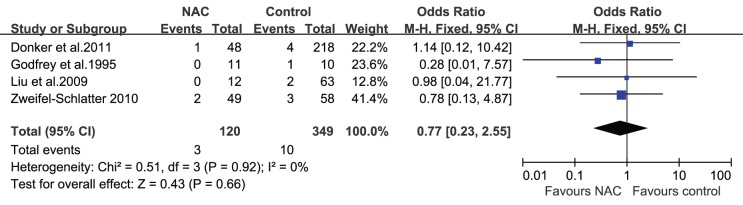
Pooled results of seromas in patients treated with NAC. The size of the solid squares is inversely proportional to the variance of the study estimate. The diamond represents the fixed effects odds ratio and 95% confidence interval.

**Figure 5:**
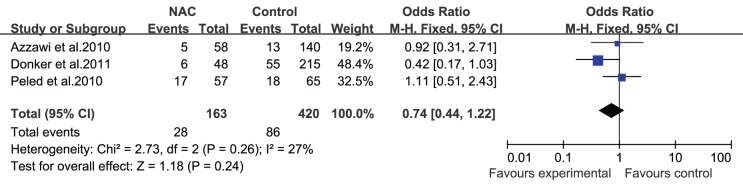
Pooled results of infections in patients treated with NAC. The size of the solid squares is inversely proportional to the variance of the study estimate. The diamond represents the fixed effects odds ratio and 95% confidence interval.

**Figure 6:**
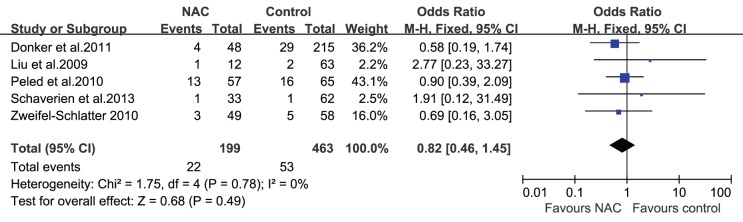
Pooled results of reoperations in patients treated with NAC. The size of the solid squares is inversely proportional to the variance of the study estimate. The diamond represents the fixed effects odds ratio and 95% confidence interval.

The studies included in this review reported different wound complications such as fat necrosis, wound dehiscence, hematoma, and infection. Azzawi et al. [22] demonstrated that minor complications (wound infection, slow healing, wound breakdown, and clinical fat necrosis) and major complications (flap loss, partial flap necrosis, hematoma, implant infection, wound breakdown, and pulmonary embolism) occurred at similar rates in patients who received NAC and those who did not (minor complications: 10% vs. 6%, respectively, P = 0.38; major complications: 9% vs. 9%, respectively, P = 1.0). Hematomas, seromas, and infections were reported in most studies; none of the studies revealed an association between NAC and the occurrence of hematomas, seromas, or infections after surgery. However, a study by Decker et al. [17] did reveal a trend toward increased wound complications in patients who received NAC and underwent mastectomies with immediate reconstruction, although it was not statistically significant (OR = 1.58; 95% CI = 0.98–2.58). For the 5 studies (n = 765 patients) that reported the influence of NAC on the occurrence of hematomas after surgery [9], [11], [21]–[23], the pooled result showed that there was no statistically significant difference in the incidence of hematomas between the 2 groups (OR = 1.35; 95% CI = 0.57–3.21; Fig. 3). Similarly, whether or not the patients received NAC did not affect the incidence of seromas (OR = 0.77; 95% CI = 0.23–2.55; Fig. 4) and infections (OR = 0.82; 95% CI = 0.46–1.45; Fig. 5) after immediate breast reconstruction. Three studies that examined the impact of NAC on the reoperation rate presented consistent results; all 3 studies demonstrated that NAC did not increase the reoperation rate. The pooled data in our meta-analysis of reoperation yielded similar results (OR = 0.74; 95% CI = 0.44–1.22; Fig. 6).

### NAC and Reconstruction Outcomes

**Figure 7:**
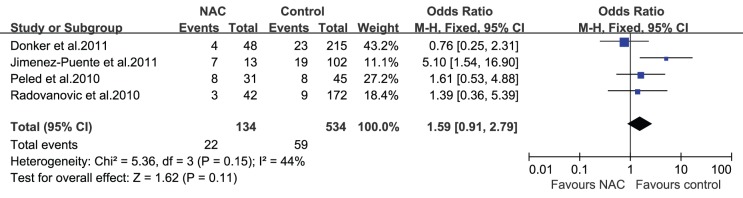
Pooled results of E/I loss in patients treated with NAC. The size of the solid squares is inversely proportional to the variance of the study estimate. The diamond represents the fixed effects odds ratio and 95% confidence interval.

Of the included reports, 6 studies [9], [18], [20], [21], [23], [28] described reconstruction outcomes including E/I loss during E/I reconstruction and total flap loss during autologous reconstruction. For E/I loss, 4 studies [9], [18], [20], [21] provided mixed results. Three studies [9], [20], [21] revealed no NAC-related increase in the risk of E/I loss in women who underwent E/I reconstruction. However, Jiménez-Puente et al. [18] observed a markedly elevated E/I loss rate (OR = 5.10, P = 0.004) in women who received NAC (7/13 patients with E/I loss) compared to women who did not receive NAC (19/102 patients). The results of our pooled meta-analysis found that NAC was not associated with increased E/I loss (OR = 1.59; 95% CI = 0.91–2.79; Fig. 7). Exclusion of the study by Jiménez-Puente et al. [18], which had a lower quality score than the other studies, led to the lowest pooled OR (OR = 1.15; 95% CI = 0.59–2.24), and exclusion of the study by Donker et al. [9] resulted in the highest estimate (OR = 2.22; 95% CI: 1.12–4.40). Two studies [23], [28] that reported the impact of NAC on total flap loss provided consistent results; both studies demonstrated that NAC did not lead to an increase in the incidence of total flap loss after autologous reconstruction.

### NAC and Start of Adjuvant Therapy

Three studies [22], [23], [28] provided information about whether NAC delayed adjuvant therapy after immediate breast reconstruction. Azzawi et al. [22] reported the interval between breast reconstruction and adjuvant radiotherapy for 67 patients (39 treated with NAC and 28 not treated with NAC). The fraction of patients with a delayed adjuvant therapy start time did not significantly differ between the 2 groups (4/39 NAC-treated patients vs. 3/28 control patients; P = 1.00). A prospective study by Zweifel-Schlatter et al. [23] also reached a similar conclusion. However, these were small, single-center studies; therefore, the results are somewhat unconvincing. Future large, multicenter studies are needed to study this issue.

### Publication Bias

**Figure 8:**
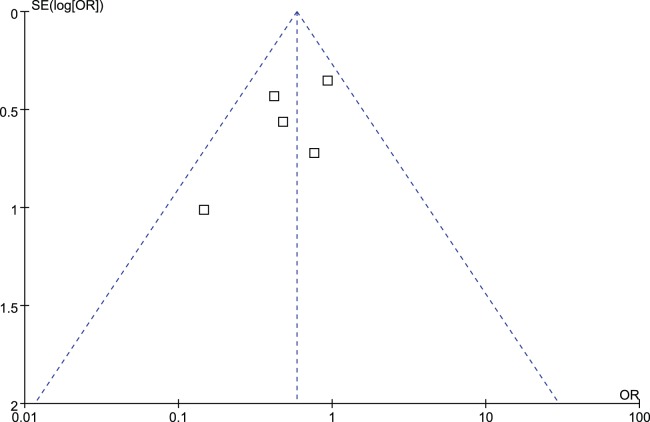
Funnel plot demonstrating the absence of publication bias among the studies that reported overall complications.

Figure 8 shows that the plots are relatively symmetric, which means that there is no significant publication bias in the reports of the overall complications. However, because of the limited number of included studies included in the analysis, publication bias might be inevitable.

## Discussion

This is the first meta-analysis to examine the impact of NAC on immediate breast reconstruction. Our meta-analysis demonstrated that patients treated with NAC were less likely to suffer negative outcomes such as wound complications (hematomas, seromas, and infections) and reconstruction failure (E/I loss) after immediate breast reconstruction when compared with patients who did not receive NAC. The meta-analysis estimates were robust across sensitivity analyses that accounted for reconstruction type, follow-up period, and study quality.

NAC can kill proliferative cells, and it is known to have adverse effects on the immune system, which might cause a higher complication rate after surgery. We unexpectedly uncovered the interesting phenomenon that patients who received NAC actually had lower complication rates after breast reconstruction. Notably, a meta-analysis of the impact of NAC on breast surgery performed by Mieog et al. [12] reached a similar conclusion. However, it is unclear why improved outcomes were observed in patients who received NAC. Patient selection bias might have contributed to this phenomenon. In certain studies, the patients in the NAC-treated group who were selected for immediate breast reconstruction were younger and had fewer comorbidities than patients in the control group. The experience and operation skills of the surgeon are also very important factors that could influence early surgical complications. Therefore, it is inappropriate to conclude that NAC can reduce complication rates. However, we can say that patients treated with NAC who are selected by their surgeons as good candidates for immediate breast reconstruction can be expected to have good postoperative outcomes.

Our pooled estimates of the occurrence rates of hematomas, seromas, and infections did not indicate an association between NAC and an increased rate of wound complications, although most of the included individual studies did see an association. We also identified 2 studies that described slightly different results. Decker et al. [17] found a trend toward increased complications in patients treated with NAC who underwent mastectomies with immediate reconstruction, although the trend was not statistically significant (OR = 1.58; 95% CI = 0.98–2.58). In their study, they considered superficial surgical site infections, deep infections, and wound dehiscence to be wound complications, which was different from our criteria for complications. Additionally, their data from axillary dissection and radiotherapy was unclear. They also limited their follow-up time to 30 days, which was shorter than that of some of the other included studies, and this might have contributed to the differences. Moreover, Mehrara et al. [24] presented data as a retrospective review of 952 patients undergoing microvascular reconstruction, and they showed that patients treated with chemotherapy prior to immediate breast reconstruction had a significantly higher incidence of fat necrosis complications. They also did not find any association between NAC and flap loss, partial flap loss, or microvascular complications. This phenomenon might be explained by the complexity of microvascular breast reconstruction, as fat necrosis was more common in this procedure. Although our study findings suggest that NAC is not associated with wound complications after immediate breast reconstruction, it is premature to reach this conclusion. Therefore, further research is needed on this topic.

The pooled estimates in our analysis demonstrated that the E/I loss rate did not increase in patients who received NAC. However, when we performed a sensitivity analysis, we found that the OR became statistically significant if the study by Donker et al. [9] was excluded. The study by Donker et al. was determined to be of high quality; therefore, this result was not very convincing. Therefore, it seems likely that NAC did not increase the E/I loss rates. However, the sample size was small; therefore, we recommend future studies on this issue. Only 2 studies provided data about autologous reconstruction, and both studies did not find any association between NAC and total flap loss. However, it was not possible to reach a firm conclusion. Further studies need to be performed to clarify this issue.

The guiding goal of immediate breast reconstruction is to provide better aesthetic results and improved quality of life. From the time immediate breast reconstruction was first introduced as part of breast cancer treatment, this procedure has gained popularity, and it has been shown to improve quality of life. Many studies have supported the oncological and surgical safety of this procedure [4]–[6]. Some surgeons hold a conservative attitude toward immediate breast reconstruction in patients treated with NAC. Our analysis supported the conclusion that patients treated with NAC can be expected to have good postoperative outcomes after undergoing immediate breast reconstruction. This finding suggests that patients receiving NAC who are selected by their doctors as good candidates can safely undergo immediate breast reconstruction. However, NAC should not be the only factor considered when determining whether a patient is a good candidate for immediate breast reconstruction; other factors including age, body mass index (BMI), and diabetes mellitus are also risk factors that might lead to higher complication rates and even failure of reconstruction. Jiménez-Puente et al. [18] analyzed 118 patients who underwent immediate breast reconstruction after mastectomies, and they found that the age of the patients was related to the risk of reconstruction failure (OR = 3.02, P = 0.02). Decker et al. [17] performed a logistic analysis of 44,533 breast surgeries by using a multivariable model to explore factors associated with wound complications. They found that smoking, obesity, and diabetes mellitus were predictive factors of wound complications after breast surgery (smoking: OR = 1.56, P<0.0001; obesity: OR = 2.16, P<0.0001; diabetes mellitus: OR = 1.57, P<0.0001). Therefore, consideration of variables other than NAC treatment is very important when determining whether patients should undergo immediate breast reconstruction.

Certain limitations were inevitable in this analysis. First, we did not consider certain confounding factors (such as BMI and age), the different NAC regimens used, and the interval between NAC and surgery, because the original data for all these was unavailable. These factors might affect the rate of complications after reconstruction. Second, selection bias, especially the tendency to administer NAC to patients with later-stage tumors, was a particular problem in most of the studies. Third, differences in the follow-up periods may also limit interpretation of the results. Lastly, all the studies in our analysis were non-RCTs, the results of which are not as convincing as those of RCTs. All these limitations might affect, to a certain extent, the validity of our conclusions.

## Conclusions

This meta-analysis suggests that NAC does not increase the complication rate after immediate breast reconstruction. Immediate breast reconstruction following NAC treatment was a safe procedure for appropriately selected patients. However, the majority of studies were performed via retrospective analysis to examine small numbers of patients at single centers. Therefore, we believe that a multicenter prospective study with a longer follow-up period and more clearly defined parameters would be the best way to study this issue in the future.

## Supporting Information

Checklist S1 **PRISMA Checklist.**
(DOC)
